# University students’ alcohol use behaviour and self-efficacy to abstain from alcohol use: data from Ghana

**DOI:** 10.1186/s13104-019-4695-0

**Published:** 2019-10-16

**Authors:** Franklin N. Glozah, Joyce Komesuor

**Affiliations:** 1 Department of Social and Behavioural Sciences, School of Public Health, University of Ghana, Accra, Ghana; 2 Department of Population and Behavioural Sciences, School of Public Health, University of Health and Allied Sciences, Ho, Ghana

**Keywords:** Alcohol use, Alcohol use self-efficacy, Psychometrics, Ghana, University students

## Abstract

**Objectives:**

The university students’ alcohol use behaviour and alcohol use self-efficacy data were collected among Ghanaian university students. This survey had two main objectives: (a) to examine the factorial validity, structure, and reliability of the 20-item alcohol abstinence self-efficacy scale among undergraduate students and (b) to examine the role of alcohol abstinence self-efficacy in the pros and cons of alcohol use. These two objectives have been published as separate research articles without the data (including data management) that were used for analyses. As the data are not already available as part of these published papers, this data note provides a composite and integrated data grounded on these published research articles with detailed information on the instruments used to collect data, raw data in statistical software, managed/coded data in statistical software, and generated correlation matrix used to perform complex analysis.

**Data description:**

The data includes information on two major dimensions: (a) alcohol abstinence self-efficacy—negative affect, social interactions and positive states, physical pain/illness, and alcohol craving (thoughts about using) and (b) decisional balance—measuring the benefits (pros) of alcohol use and the costs (cons) of alcohol use. In addition, data were collected on socio-demographic characteristics of students including their age, sex, level/year in school, stream of study, place of residence and religious affiliation. The data collected is more representative of students in private universities.

## Objective

There is an increasing awareness of indiscriminate alcohol use among University students world-wide. Self-efficacy to abstain from alcohol use is known to play a significant role in alcohol use abstinence by perceiving fewer benefits (pros) and perceiving more costs (cons) in alcohol use. However, not much is known about self-efficacy to abstain from alcohol use in relation to the pros and cons of alcohol use among Ghanaian university students. This survey had to main objectives: (a) to examine the factorial validity, structure, and reliability of the 20-item alcohol abstinence self-efficacy scale in undergraduate students and (b) to examine the role of alcohol abstinence self-efficacy in the pros and cons of alcohol use. The survey was conducted due, primarily, to the fact that there is a paucity of research literature documenting alcohol use behaviour and alcohol abstinence self-efficacy in Ghanaian university students. Two major publications have resulted from these two main objectives. These are [1] that examined the factorial validity, structure, and reliability of the 20-item alcohol abstinence self-efficacy scale in undergraduate students and [2] that examined the role of alcohol abstinence self-efficacy in the pros and cons of alcohol use in undergraduate students. As the data are not already available as part of these published papers, this data note provides a composite and integrated data grounded on these published research articles with detailed information on the instruments used to collect data, raw data in statistical software, managed/coded data in statistical software, and generated correlation matrix used to perform complex analysis. Furthermore, this data note is meant to serve as benchmark data particularly for Ghana and Sub-Saharan Africa in general, due to the paucity of research literature and data documenting alcohol use behaviour and alcohol abstinence self-efficacy among university students within these contexts.

## Data description

The data handling was identical to our publication [1] from 2015 that consisted of 215 undergraduate students from a population of about 2000 students in a private university in Ghana. A simple random sampling method was used to select a few classes from various programs. All participants gave a written informed consent before participating in the study.

In addition to pertinent socio-demographic characteristics, two main instruments were used for data collection—the alcohol abstinence self-efficacy scale and alcohol decisional balance scale. The alcohol abstinence self-efficacy scale [3] assesses self-efficacy and evaluates an individual’s efficacy to abstain from drinking in 20 situations that represent typical drinking cues. Like our 2015 publication [1], participants were asked to give a current estimate of their efficacy to abstain from alcohol. These situations constituted four sub-scales and are rated on a 5-point Likert scale, where higher scores indicate higher self-efficacy to abstain from alcohol use.

The alcohol decisional balance scale consists of 10 items measuring the benefits (pros) of alcohol use and 10 items measuring the costs (cons) of alcohol use [4]. Participants responded on a 5-point Likert scale ranging from 0—not at all important to 4—extremely important on both the pros and cons subscales with higher scores representing both more pros and cons of drinking alcohol. In populations or cultures with less drinking experience, the measure is commonly construed as an individual’s decision making regarding whether or not to drink alcohol at all [4]. Items for both alcohol abstinence self-efficacy scale and alcohol decisional balance scale are available on Harvard dataverse [5]. Table 1 contains a link to Harvard dataverse (Data file 1) where the questionnaire used for data collection could be retrieved.

**Table 1:** Overview of data files

Label	Name of data file	File types (file extension)	Data repository and identifier (DOI or accession number)
Data set 1	Correlation matrix	AASESmx.tab	Harvard Dataverse (10.7910/DVN/6R4DOG) [5]
Data set 2	SPSS raw data	Raw.tab	Harvard Dataverse (10.7910/DVN/6R4DOG) [5]
Data set 3	SPSS coded data	Scales.tab	Harvard Dataverse (10.7910/DVN/6R4DOG) [5]
Data file 1	Questionnaire	Research Questionnaire.pdf	Harvard Dataverse (10.7910/DVN/6R4DOG) [5]

In addition, Table 1 contains information about the raw data (Data set 2)—data entered into SPSS directly from completed questionnaires, and coded data (Data set 3)—data in SPSS with various scales and subscales created. Furthermore, Table 1 contains information on the correlation matrix (Data set 1) that was generated and used in AMOS software to perform structural equation modelling to examine the factorial validity of the instruments. A correlation matrix shows correlation coefficients between variables of interest—each cell in the table (Data set 1) shows the correlation between two variables, used to summarize a large dataset for further statistical analysis.

## Limitations

First, a relatively small sample size was used in this survey so generalizing the data (collected from students in a single private university) to the whole of Ghana should be done with caution. However, although the sample size was relatively small, power analysis for a MANCOVA with eight groups, four predictors and two dependent variables conducted in G-POWER [6] to determine a sufficient sample size using an alpha of 0.05, a power of 0.90, and a small effect size of 0.10 produced a desired sample size of 100, suggesting that the use of 215 participants has most likely produced quality data. Secondly, there were more non-drinkers compare to drinkers in the sample and this could have biased the significance of the findings. Finally, the questionnaires only gathered data with respect to normal alcohol use—the results could have been different if data had been gathered on heavy alcohol use among university students.

## Data Availability

The data described in this Data note can be freely and openly accessed on Harvard Dataverse 10.7910/DVN/6R4DOG. Please see Table 1 and reference list for details and links to the data.

## Abbreviations

AASES: alcohol abstinence self-efficacy scale
ADBS: alcohol decisional balance scale
